# Perforated Meckel’s Diverticulum in a Neonate With Bucket‐Handle Anorectal Malformation: A Rare Surgical Emergency

**DOI:** 10.1155/crpe/2238981

**Published:** 2026-02-14

**Authors:** Umar Mahmood, Rija Khalid, Chaudhary Ehtsham Azmat, Wajeeh Ur Rehman, Sunaina Siddique, Raisa Bakhat

**Affiliations:** ^1^ Pediatric Surgery, The Children Hospital and University of Child Health Sciences, Lahore, Pakistan

**Keywords:** anorectal malformation, bucket-handle deformity, intestinal perforation, Meckel’s diverticulum, neonatal pneumoperitoneum

## Abstract

**Background:**

Neonatal pneumoperitoneum is a life‐threatening condition requiring urgent surgical intervention. Although Meckel’s diverticulum (MD) and anorectal malformations (ARMs) are individually recognised congenital anomalies, their coexistence is uncommon, and perforated MD in a neonate with a bucket‐handle ARM has been rarely described in the literature.

**Case Presentation:**

A term male neonate (birthweight 2600 g) presented on Day 4 of life with progressive abdominal distension and nonbilious vomiting. Examination revealed tachycardia, tachypnoea, delayed capillary refill and a bucket‐handle ARM with meconium staining. Abdominal radiograph showed free subdiaphragmatic air. Emergency laparotomy identified a 4‐5 mm perforation at the tip of a MD located 40 cm proximal to the ileocaecal valve, with severe feculent contamination. A wedge diverticulectomy including the perforated segment was performed, the ileum was closed transversely in two layers and a divided sigmoid colostomy was fashioned. Recovery was uneventful.

**Discussion:**

Perforation occurs in only 3%–10% of symptomatic MD cases and is extremely rare in neonates, particularly with coexisting ARM. No prior literature describes perforated MD in a bucket‐handle ARM. This case highlights the importance of thorough evaluation for associated gastrointestinal anomalies in ARM patients and raises the question of whether selective or routine screening is warranted.

## 1. Introduction

Neonatal pneumoperitoneum is a life‐threatening emergency that typically results from necrotising enterocolitis, spontaneous intestinal perforation or congenital anomalies of the gastrointestinal tract [1].

Meckel’s diverticulum (MD), the most common congenital anomaly of the small intestine, has a prevalence of 0.3%–2.9% and may present with complications including bleeding, obstruction and, less commonly, perforation [2–4].

Anorectal malformations (ARMs) represent a broad spectrum of congenital anomalies, with a higher prevalence and greater clinical burden in low‐ and middle‐income settings [5]. The bucket‐handle deformity is an uncommon low‐type ARM usually identified at birth [6].

Although MD and ARM may individually occur in neonates, their coexistence is rare. Only isolated reports describe MD incidentally associated with ARM, and none document perforated MD in a neonate with a bucket‐handle ARM [7].

This report describes an unprecedented combination of these anomalies and highlights the importance of maintaining a high index of suspicion for associated gastrointestinal pathology in neonates presenting with ARM.

## 2. Case Presentation

A term male neonate, birthweight 2600 g, born via spontaneous vaginal delivery at a peripheral healthcare facility, presented to the Neonatal Emergency Unit on Day 4 of life with progressive abdominal distension and multiple episodes of nonbilious, nonprojectile vomiting over two days. He had not passed stool during the first 48 h of life, although a small amount of meconium staining was noted on Day 3. The parents reported noticing an abnormal perineal appearance but initially sought care at a local clinic, contributing to the delayed presentation of more than 72 h.

On arrival, the neonate appeared lethargic and dehydrated. Vital parameters were as follows: heart rate 168/min, respiratory rate 58/min, temperature 36.7°C, oxygen saturation 92% on room air and capillary refill time  > 3 s. Initial resuscitation included warming, supplemental oxygen, nasogastric tube decompression, Foley catheterisation, intravenous fluids and broad‐spectrum antibiotics.

**FIGURE 1:**
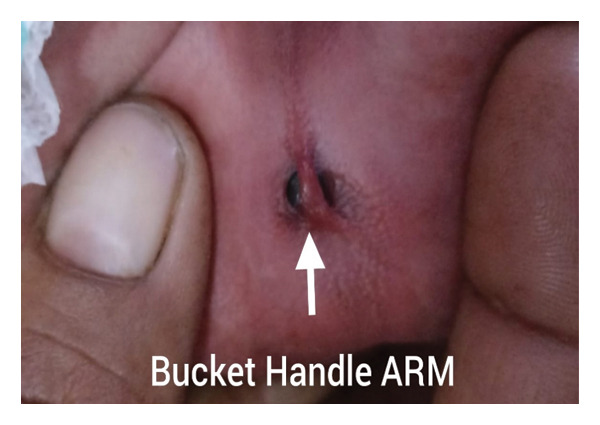
Perineal photograph showing the bucket‐handle anorectal malformation. The arrow indicates the prominent bucket‐handle skin tag with meconium staining around the anal verge.

**FIGURE 2:**
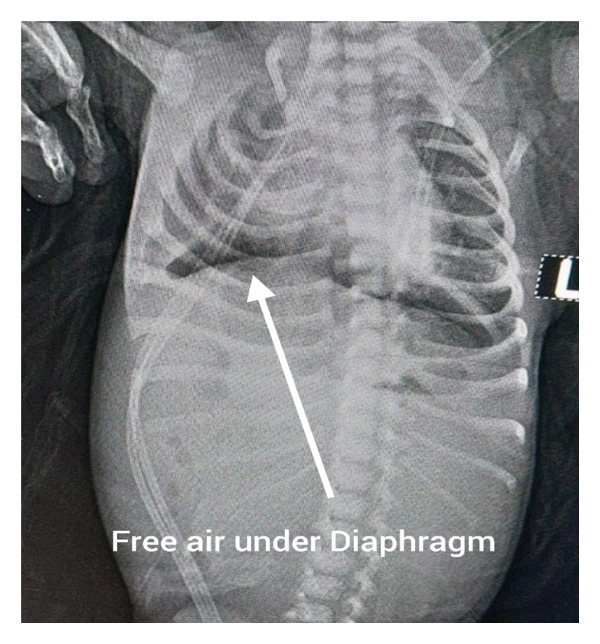
Erect abdominal radiograph demonstrating pneumoperitoneum. The arrows highlight free subdiaphragmatic air under both hemidiaphragms, consistent with intestinal perforation.

Abdominal examination revealed a tense, distended abdomen with absent bowel sounds. Perineal examination showed a bucket‐handle ARM with meconium staining around the anal verge (Figure 1). An erect abdominal radiograph demonstrated free subdiaphragmatic air, confirming pneumoperitoneum and suggesting intestinal perforation (Figure 2). With a provisional diagnosis of intestinal perforation in association with a bucket‐handle ARM, the neonate was taken for emergency exploratory laparotomy following stabilisation.

**FIGURE 3:**
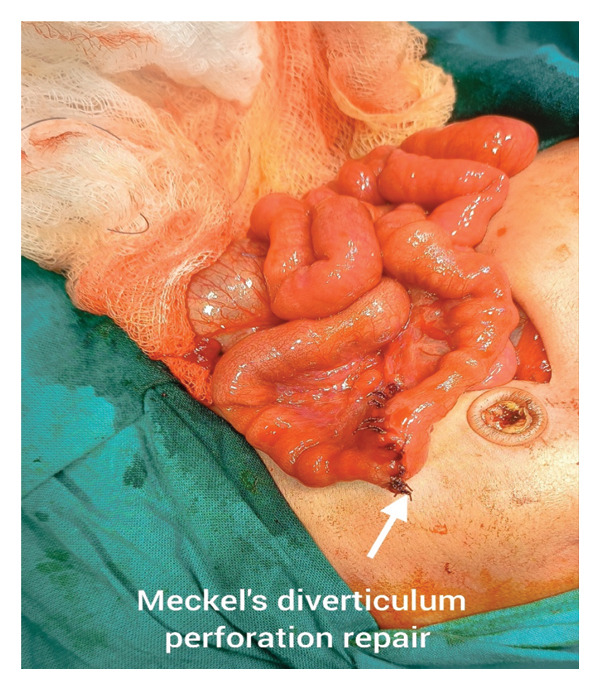
Intraoperative image showing the perforated Meckel’s diverticulum. The arrow points to the 4‐5 mm perforation at the tip of the diverticulum, located approximately 40 cm proximal to the ileocaecal valve.

Operative findings included markedly dilated bowel loops containing meconium and a 4‐5 mm perforation located at the tip of a MD, approximately 40 cm proximal to the ileocaecal valve, with severe feculent contamination throughout the peritoneal cavity. A wedge diverticulectomy including the perforated segment was performed. The resulting ileal defect was closed transversely in two layers. The resected edges were sent for histopathological examination. Given the gross contamination and the presence of ARM, a divided sigmoid colostomy was fashioned (Figure 3).

Postoperative management was provided in the Surgical Neonatal Intensive Care Unit (SNICU). The neonate remained nil per oral for 36 h, after which he began passing meconium through the stoma, allowing gradual initiation of enteral feeds. Postoperative investigations included daily complete blood counts, serum electrolytes, abdominal radiograph on postoperative Day 2 and full VACTERL screening, all of which were normal. Intravenous piperacillin–tazobactam, amikacin and metronidazole were administered for four days.

The infant was discharged on postoperative Day 4 on full feeds with a functional stoma. Histopathological examination confirmed inflamed diverticular tissue without ectopic gastric or pancreatic mucosa. At one‐week follow‐up, the neonate demonstrated good weight gain, a healthy stoma and a well‐healed laparotomy wound. Parents were counselled on stoma care, and plans were made for definitive anoplasty and subsequent colostomy reversal during follow‐up.

## 3. Discussion

Neonatal intestinal perforation is a serious surgical emergency with high morbidity and mortality, particularly in settings of delayed diagnosis or inadequate access to specialised care [8]. Pneumoperitoneum on radiography remains a key diagnostic indicator [1]. In the present case, early recognition of perforation and timely surgical intervention contributed to the favourable outcome.

MD is the most common congenital anomaly of the small intestine and becomes symptomatic in a minority of patients. Perforation occurs in only 3%–10% of symptomatic cases [9]. Neonatal perforation of MD is particularly uncommon and has been described primarily in low birthweight or premature neonates, often without associated anomalies [10, 11]. Reports of MD associated with ARMs are rare: Stoll et al. identified one such case, but no perforation was noted [7]. The coexistence of a bucket‐handle ARM and perforated MD, as seen in our case, has not been reported previously.

ARM is a common congenital anomaly in low‐ and middle‐income countries where delayed presentation is frequently encountered due to limited antenatal screening and reduced availability of specialist neonatal care [5]. The bucket‐handle variant is a low‐type ARM typically recognised shortly after birth [6]. When diagnosis is delayed, functional obstruction may occur, potentially increasing intraluminal pressure and contributing to complications such as perforation. This mechanism may explain the early occurrence of MD perforation in our patient.

Surgical management of neonatal intestinal perforation requires prompt exploration, control of contamination and restoration of intestinal integrity. For perforated MD, treatment options include wedge diverticulectomy or segmental bowel resection depending on the site and extent of involvement [10, 11]. In this case, the perforation was localised to the tip of the diverticulum, making wedge diverticulectomy appropriate. Severe feculent contamination and the associated ARM justified the creation of a diverting colostomy, with delayed definitive anorectal reconstruction planned. This staged approach aligns with accepted principles for managing ARM in the presence of peritonitis [12].

Recent regional studies from similar socioeconomic settings reinforce the challenges of delayed ARM presentation. A Pakistani tertiary‐centre study reported that ARM accounted for a significant proportion of gastrointestinal congenital anomalies and highlighted the associated morbidity in resource‐limited environments [13]. Another cross‐sectional study from Pakistan demonstrated that the majority of infants with ARM presented after 48–72 h of birth, with late presentation associated with higher complication rates [14]. Similarly, an Indian series reported delayed presentation of ARM beyond the neonatal period as a contributor to increased complications in a tertiary‐care cohort [15]. These findings mirror the delayed presentation in our patient and illustrate ongoing diagnostic challenges in comparable low‐resource settings.

This case underscores the importance of careful evaluation for concurrent gastrointestinal anomalies in neonates with ARM, especially those presenting with abdominal distension or obstructive symptoms. Although routine screening for associated gastrointestinal anomalies in all ARM cases remains debated, selected neonates with atypical symptoms or delayed presentation may benefit from targeted evaluation. To our knowledge, this is the first reported case of neonatal pneumoperitoneum caused by perforated MD in a neonate with a bucket‐handle ARM. Recognising such rare associations can guide more tailored surgical planning and may prompt reconsideration of screening practices in complex ARM presentations. [16].

A comparison of previously reported cases of MD in neonates and its association with ARM is summarised in Table 1.

**TABLE 1:** Comparison of published cases of Meckel’s diverticulum associated with neonatal or ARM presentations.

Author/year	Population	Associated ARM	MD finding	Perforation	Key notes/outcome
Stoll et al., 2007 [7]	Congenital anomalies registry	Yes (ARM; type not specified)	Incidental MD	No	Only ARM + MD association reported; no perforation
Wang et al., 2019 [10]	Very low birthweight neonate	No	MD with severe pneumoperitoneum	Yes	MD perforation due to infection/ischemia; no ARM
Liaqat et al., 2022 [11]	Six neonates	No	MD in all six cases	Yes	Largest neonatal MD perforation series; no ARM
Present case, 2024	Term neonate	Yes (bucket‐handle)	MD located 40 cm proximal to IC valve	Yes (tip perforation)	First reported coexistence of perforated MD and bucket‐handle ARM

## Funding

This work did not receive any specific funding from public, commercial or not‐for‐profit agencies. The research was conducted as part of the authors’ routine professional activities.

## Disclosure

No funding body was involved in the manuscript writing, editing, approval or decision to publish.

## Ethics Statement

Ethical approval was not required for this case report as per the Children Hospital, Lahore, Pakistan guidelines, as it involves the description of a single clinical case and does not constitute systematic research.

## Consent

Written informed consent for publication of this report, including the use of clinical details and images, was obtained from the patient’s father.

## Conflicts of Interest

The authors declare no conflicts of interest.

## Data Availability

The data that support the findings of this study are available from the corresponding author upon reasonable request.
